# Supplementary material for: Illustrating new understanding of adsorbed water on silica for inducing tetrahedral cobalt(II) for propane dehydrogenation
Source: Nat Commun. 2023 Jan 6;14:100. doi: 10.1038/s41467-022-35698-0 (PMC9823098; doi:10.1038/s41467-022-35698-0)
Supplement: Supplementary file 1 — Supplementary Information [file 41467_2022_35698_MOESM1_ESM.pdf]

**Supporting Information**

# Illustrating new understanding of adsorbed water on silica for inducing tetrahedral cobalt(II) for propane dehydrogenation

Zijun Huang[1,2,3,4], Dedong He[1,2,3,4]*, Weihua Deng[1,3,4], Guowu Jin[2,3,4], Ke Li[2,3,4], Yongming Luo[1,2,3,4]*

[1] Faculty of Environmental Science and Engineering, Kunming University of Science and Technology, Kunming 650500, P. R. China.

[2] Faculty of Chemical Engineering, Kunming University of Science and Technology, Kunming 650500, P. R. China.

[3] The Innovation Team for Volatile Organic Compounds Pollutants Control and Resource Utilization of Yunnan Province, Kunming 650500, P. R. China.

[4] The Higher Educational Key Laboratory for Odorous Volatile Organic Compounds Pollutants Control of Yunnan Province, Kunming 650500, P. R. China.

*Corresponding author, E-mail: dedong.he@qq.com (D. He); environcatalysis@kust.edu.cn (Y. Luo)

## Contents

1. Experimental Section

2. Supplementary Figures and Notes

3. Supplementary Tables

## 1. Experimental Section

**Chemicals and Materials.** Tetraethylorthosilicate (98%, Aladdin company), pluronic triblock copolymer (BASF, $EO_{20}$-$PO_{70}$-$EO_{20}$, P123) (average molecular weight 5800, Aldrich), HCl (Sinopharm Chemical Reagent Co., Ltd.), cobalt nitrate hexahydrate ($Co(NO_3)_6 \cdot H_2O$, 99.99% Aladdin company)

**Synthesis of SBA-15.** The pluronic triblock copolymer (BASF, $EO_{20}$-$PO_{70}$-$EO_{20}$, P123) was used as the structure-directing agent, and tetraethyl orthosilicate (TEOS, 98%, Aldrich) was applied as a source of silica. In a typical synthesis, the triblock copolymer was dissolved in 650 mL of deionized water, when P123 was completely dissolved, 140 mL of hydrochloric acid solution was added. Then stirring at 40 $^{o}$C for 3 h, following, 55 mL of TEOS was added dropwise and kept stirring for 24 h, and the formed gel was crystallized at 90 °C for 24 h. The washed sample was dried at 90°C for 24 h. Then, the polymer was removed by calcination in air at 550 °C for 6 h.

**Catalysts characterization.** The powder X-ray diffraction (XRD) patterns of the catalysts were measured on a Rigaku D/max-1200 diffractometer using CuKα radiation (λ =1.54 Å, 40 kV and 30 mA). Quasi *in-situ* X-ray photoelectron spectroscopy (XPS) measurement was performed on a Thermo ESCALAB 250Xi spectrometer, equipped with a monochromatic Al Kα radiation source (hv = 1486.6 eV). In the reaction chamber, the sample was reduced in 10%$H_2$/Ar at 600 $^{o}$C for 2 h. After treatment, the sample was cooled down to room temperature, and then directly transferred to analytical chamber in a vacuum (UHV XPS) to avoid exposure to air.

The peak shift caused by catalyst charging was corrected by adjusting the binding energy of the C1s peak to 284.8 eV.

$H_2$-temperature-programmed reduction ($H_2$-TPR) were carried out on a homemade instrument. The $H_2$-TPR process was conducted from 30 to 900 °C under 10% $H_2$/Ar flow (30 mL /min) with a heating rate of 10 °C/min. The $H_2$ signal was monitored by the TCD. Ultraviolet-visible (UV-vis) spectra were recorded from 200 to 800 nm on a Shimadzu2501PC with a Cary 500 spectrometer and $BaSO_4$ was used as the benchmark. Transmission electron microscopy (TEM) measurements were performed on a FEI-TALOS-F200X microscope operated at 100 kV. For TEM analysis, the sample powder was placed in absolute ethanol by an ultrasonic bath suspension and then deposited on a Formvar-coated 200 mesh Cu grid. The $^1$H NMR spectra were obtained on a Bruker 400 MHz WB Solid-State NMR spectrometer at a resonance frequency of 400.1MHz, duration of 6.5 μs and a recycle delay of 4 s. *In-situ* $NH_3$-DRIFTS were recorded using a Thermo Fisher Nicolet iS50 FT-IR spectrometer, equipped with a MCT/A detector and KBr windows, and a high temperature reaction chamber under ambient pressure. In a typical run, solid sample was loaded into the chamber and treated at 150 °C, 250 °C, 400 °C and 600 °C for 2 h in 10% $H_2$/Ar (30 mL/min). Then, the chamber was cooled to the room temperature (25 °C), and $NH_3$ (0.02% $NH_3$ in $N_2$) was flowed through the sample for 30 min. Subsequently, the chamber was heated to 150 °C for desorption under $N_2$, keeping 1 h and cooled to 25 °C, the DRIFTS signals were recorded. *In situ* FT-IR were recorded using a

BRUKER VERTEX 70 spectrometer equipped with the high temperature reaction chamber. The sample was heated to 600 °C in 10% $H_2$/Ar flow (30 mL/min) at a heating rate of 10 °C $min^{-1}$. The FT-IR signals were recorded every 10 °C.

**DFT calculation.** All of spin-polarized calculations based on density functional theory (DFT) were performed by utilizing DMol3 package. The generalized gradient approximation (GGA) in the Perdew–Burke–Ernzerhof form and Semicore Pseudopotential method (DSPP) with the double numerical basis sets plus the polarization functional (DNP) were adopted. The double numerical basis sets plus the polarization functional (DNP) were adopted. A DFT-D correction with Grimme scheme was used to account for the dispersion interaction. The SCF convergence for each electronic energy was set as $1.0 \times 10^{-5}$ Ha, and the geometry optimization convergence criteria were set up as follows: $1.0 \times 10^{-5}$ Ha for energy, 0.004 Ha Å-1 for force, and 0.01 Å for displacement, respectively. Energy barriers were examined by linear and quadratic synchronous transit methods in combination with the conjugated gradient (CG) refinement. The free energies were obtained by G = Etotal + EZPE-TS, where Etotal, EZPE, and TS are the ground-state energy, zero-point energies, and entropy terms, respectively, with the latter two taking vibration frequencies from DFT. Finally, the reaction energies (G) of different intermediates are defined as ΔG = Gi-Greactant (Ei is the energy of intermediates and Ereactant is the total energy of reactants).

## 2. Supplementary Figures and Supplementary Notes

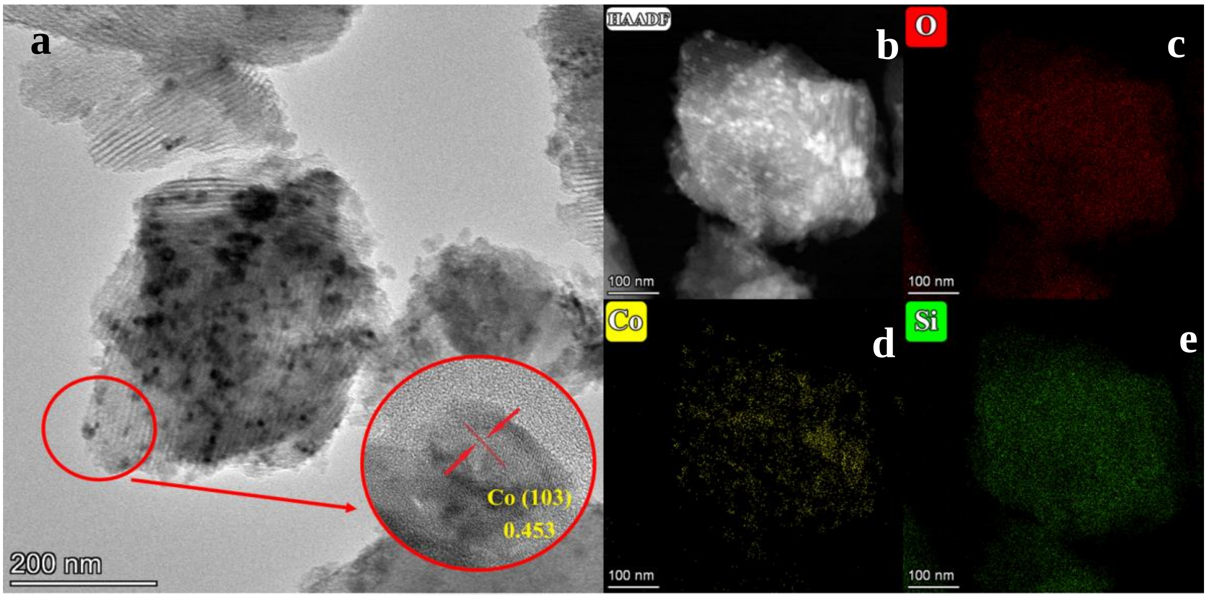


**Supplementary Fig. 1. Morphology of $H_2$-reduction catalyst**. (a) TEM image, (b) STEM image, EDS elements mapping of (c) O, (d) Co and (e) Si

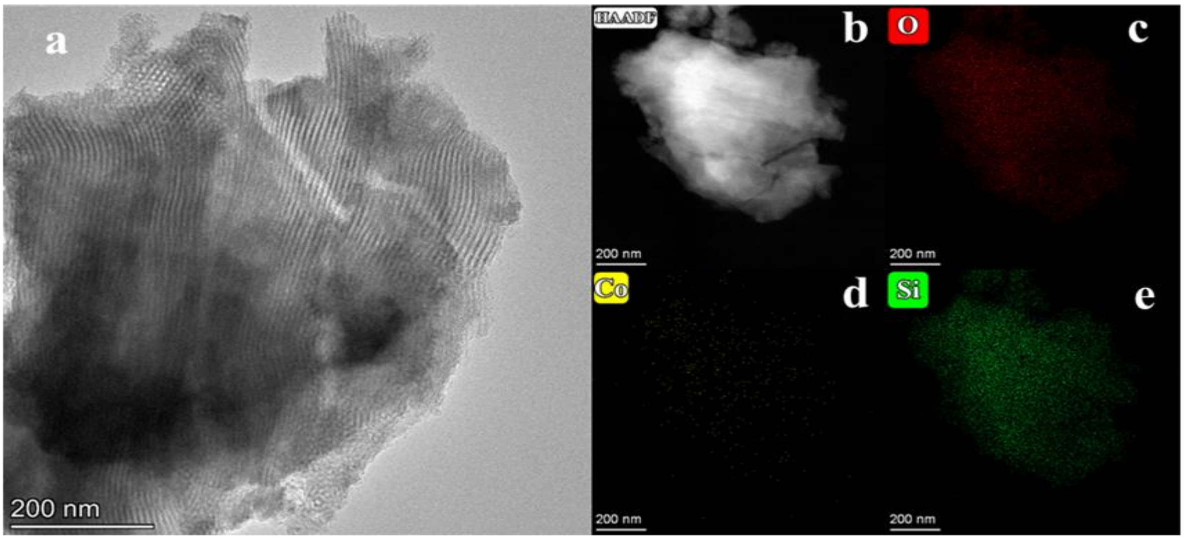


**Supplementary Fig. 2. Morphology of Dir-reduction catalyst**. (a) TEM image, (b) STEM image, EDS elements mapping of (c) O, (d) Co and (e) Si

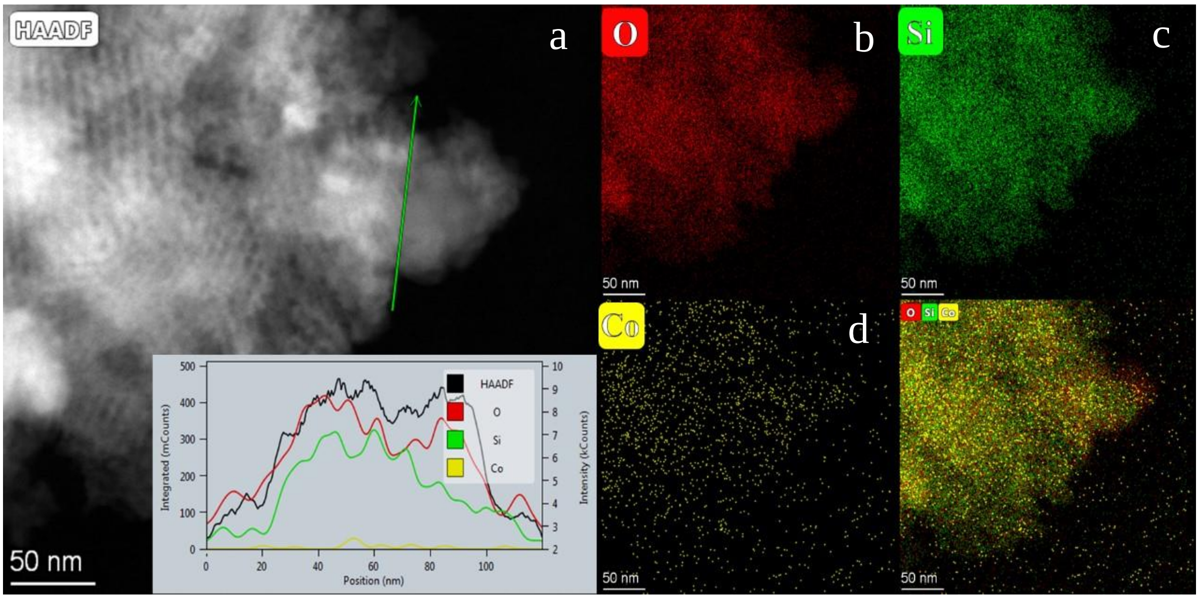


**Supplementary Fig. 3. Morphology of Dir-reduction catalyst.** (a) STEM image, EDS elements mapping of (b) O, (c) Si and (d) Co

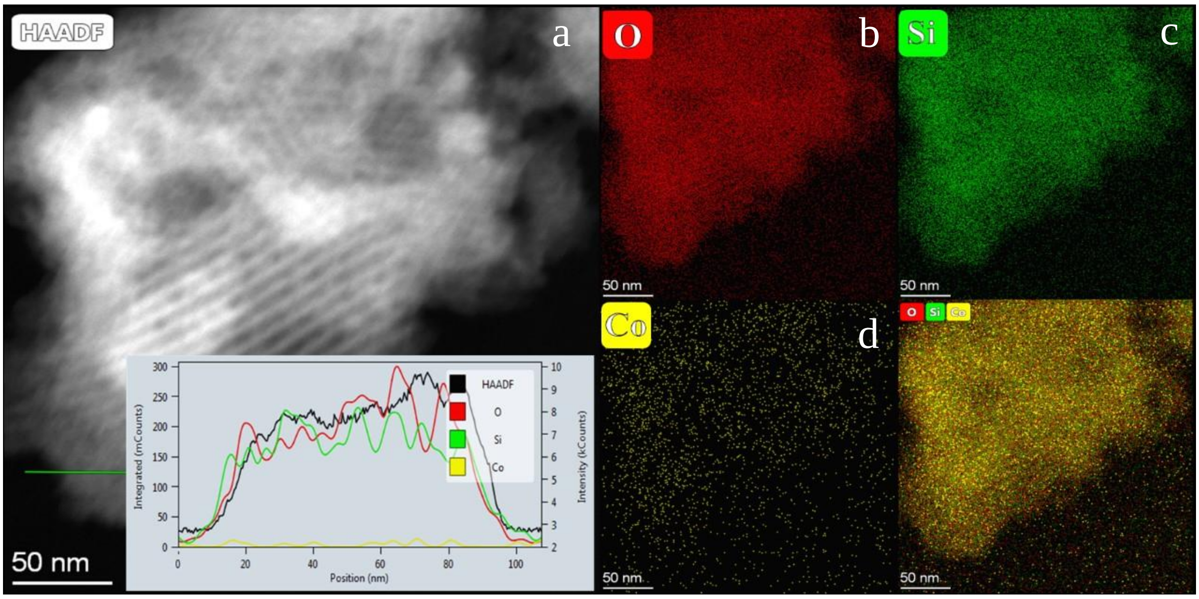


**Supplementary Fig. 4. Morphology of Dir-reduction catalyst**. (a) STEM image, EDS elements mapping of (b) O, (c) Si and (d) Co

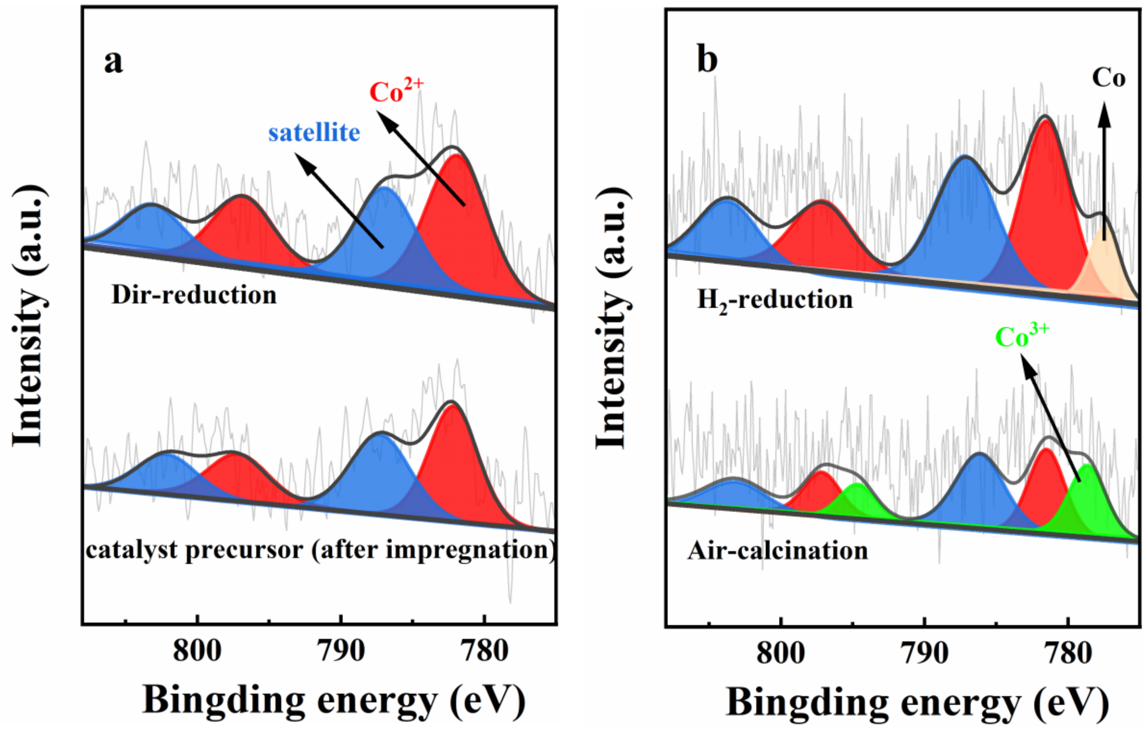


**Supplementary Fig. 5. Quasi in-situ Co 2*p* XPS spectra of** (a) Dir-reduction catalyst, (b) $H_2$-reduction catalyst.

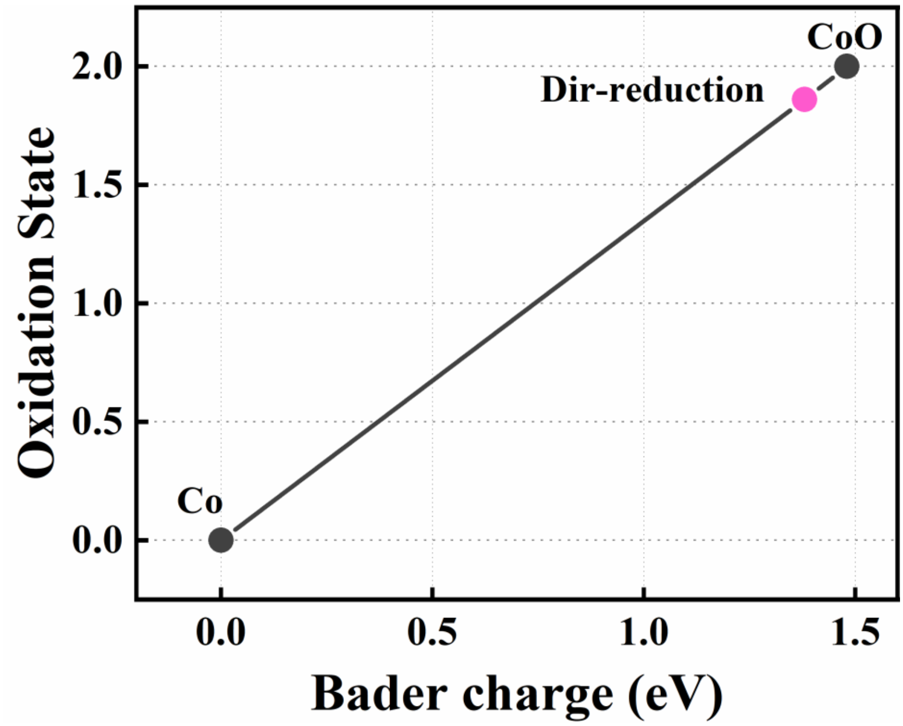


**Supplementary Fig. 6. The Bader charge and the fitted oxidation states of the Dir-reduction catalyst.**

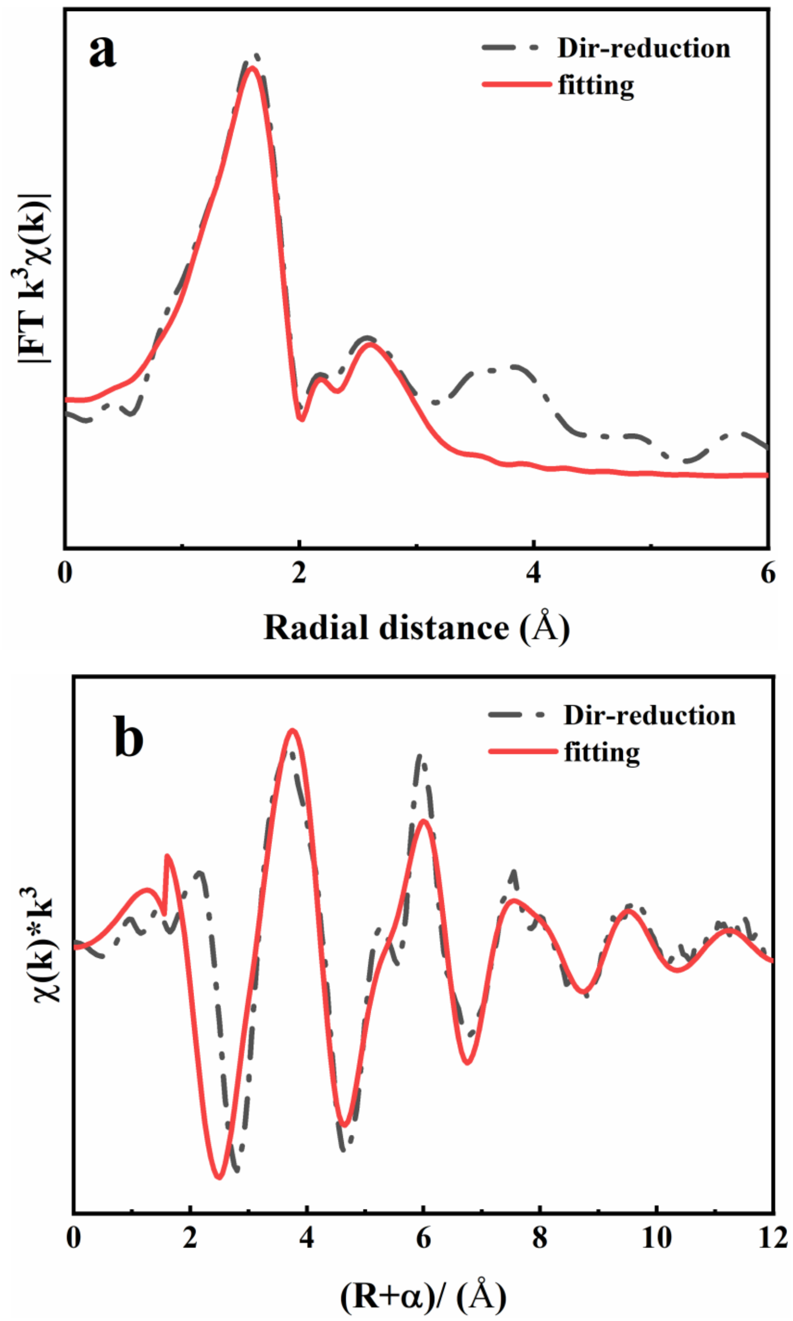


**Supplementary Fig. 7. Fourier transform of the EXAFS spectra.** (a) Fourier transform (FT) $k^3$-weighted $\chi$(k)-function of the EXAFS spectra, and (b) R-space fitting curves for Dir-reduction catalyst.

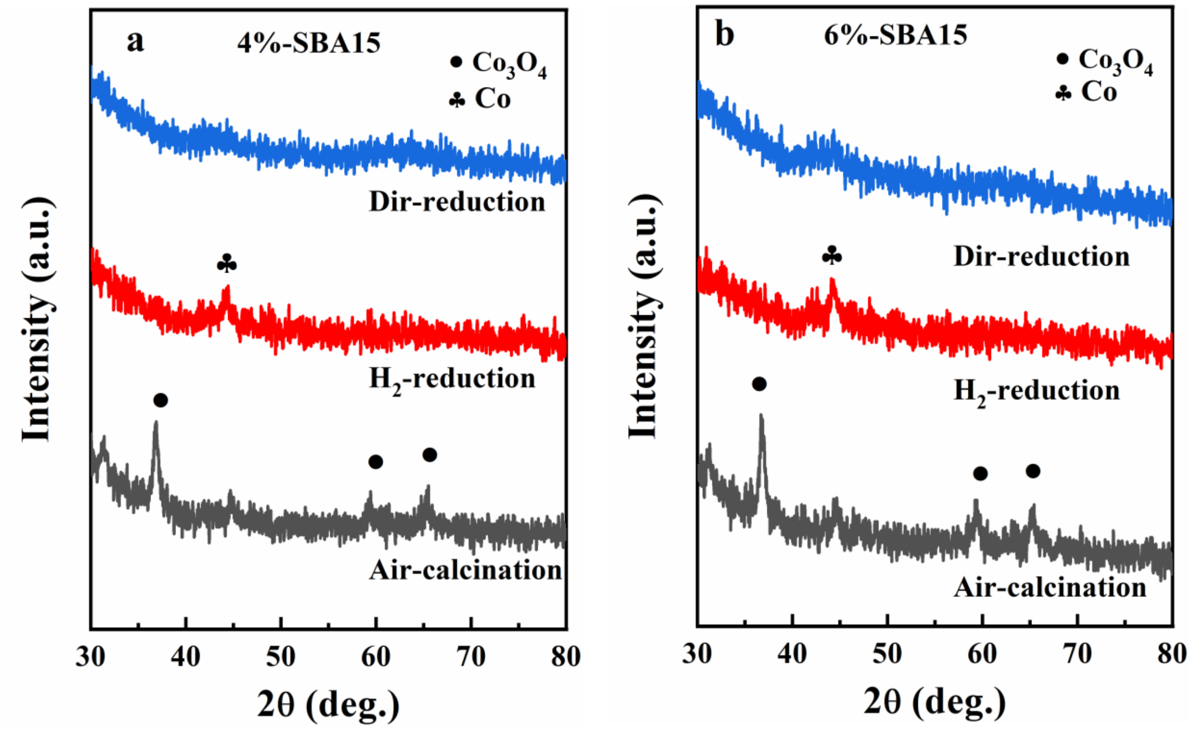


**Supplementary Fig. 8. XRD patterns** of (a) 4% Co-SBA-15, (b) 6% Co-SBA-15.

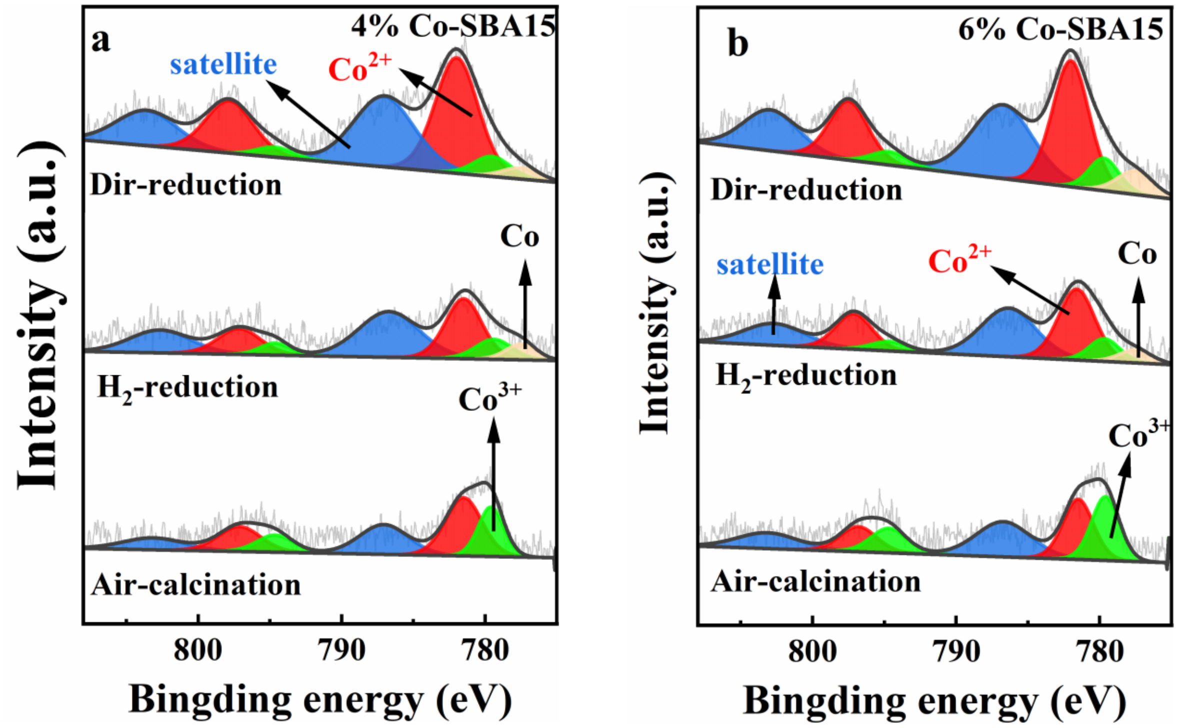


**Supplementary Fig. 9. Ex-situ XPS Co 2*p* spectra** of (a) 4% Co-SBA-15, (b) 6% Co-SBA-15.

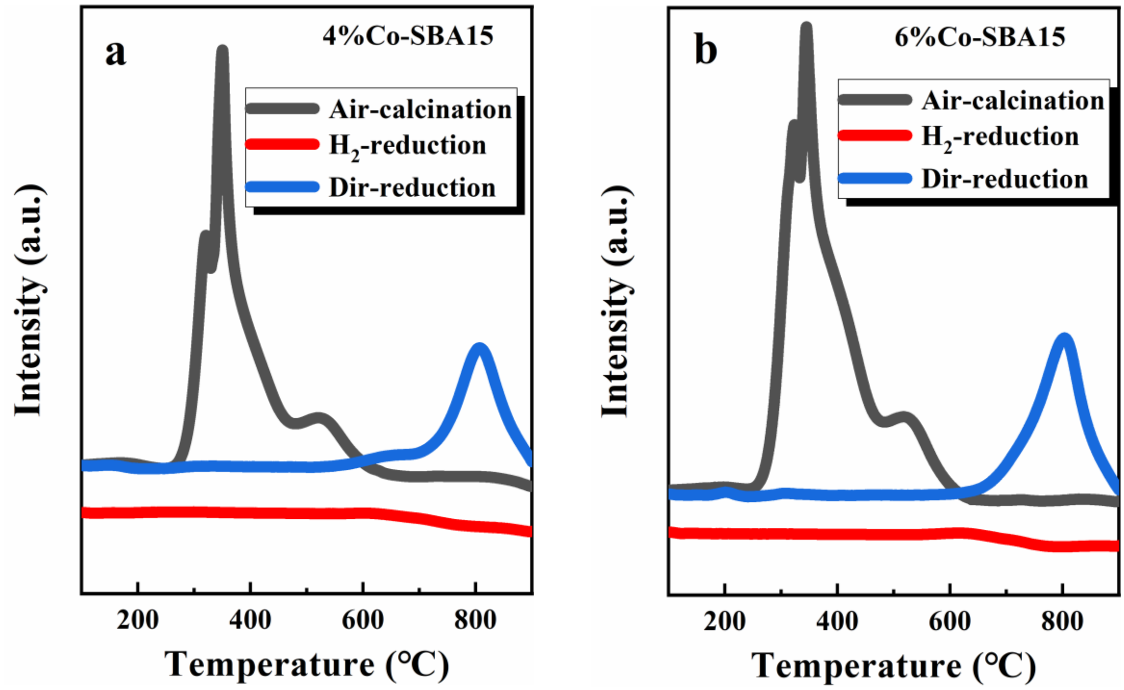


**Supplementary Fig. 10. In-situ $H_2$-TPR** of (a) 4% Co-SBA-15, (b) 6% Co-SBA-15.

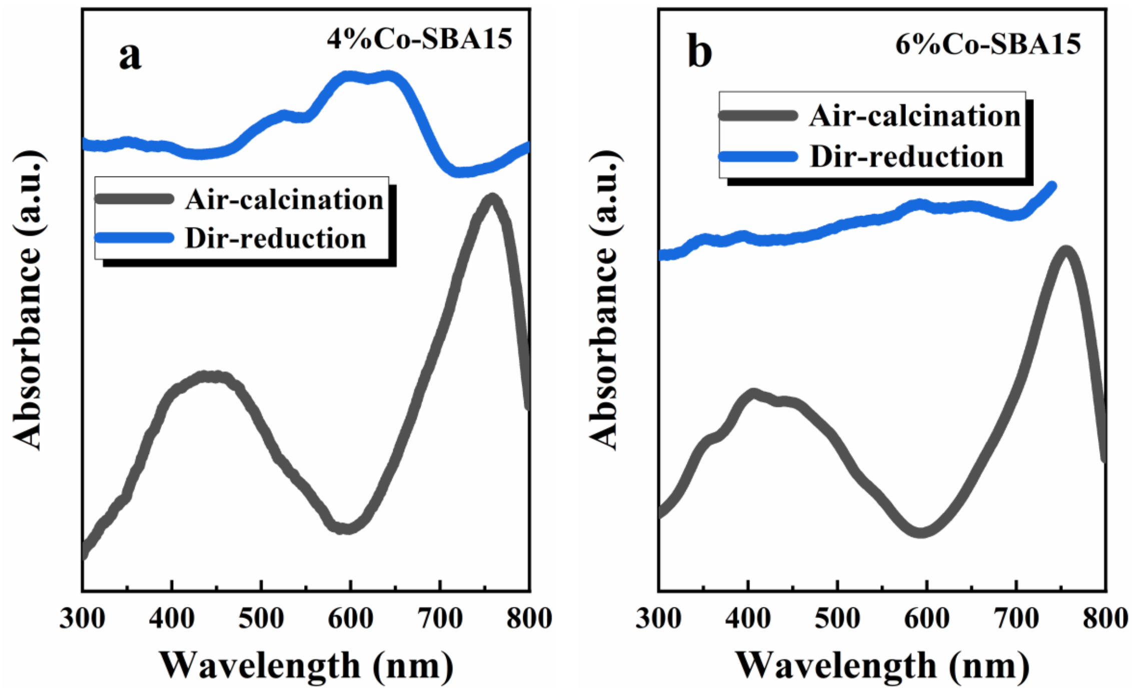


**Supplementary Fig. 11. Ex-situ UV-vis spectra** of (a) 4% Co-SBA-15, (b) 6% Co-SBA-15.

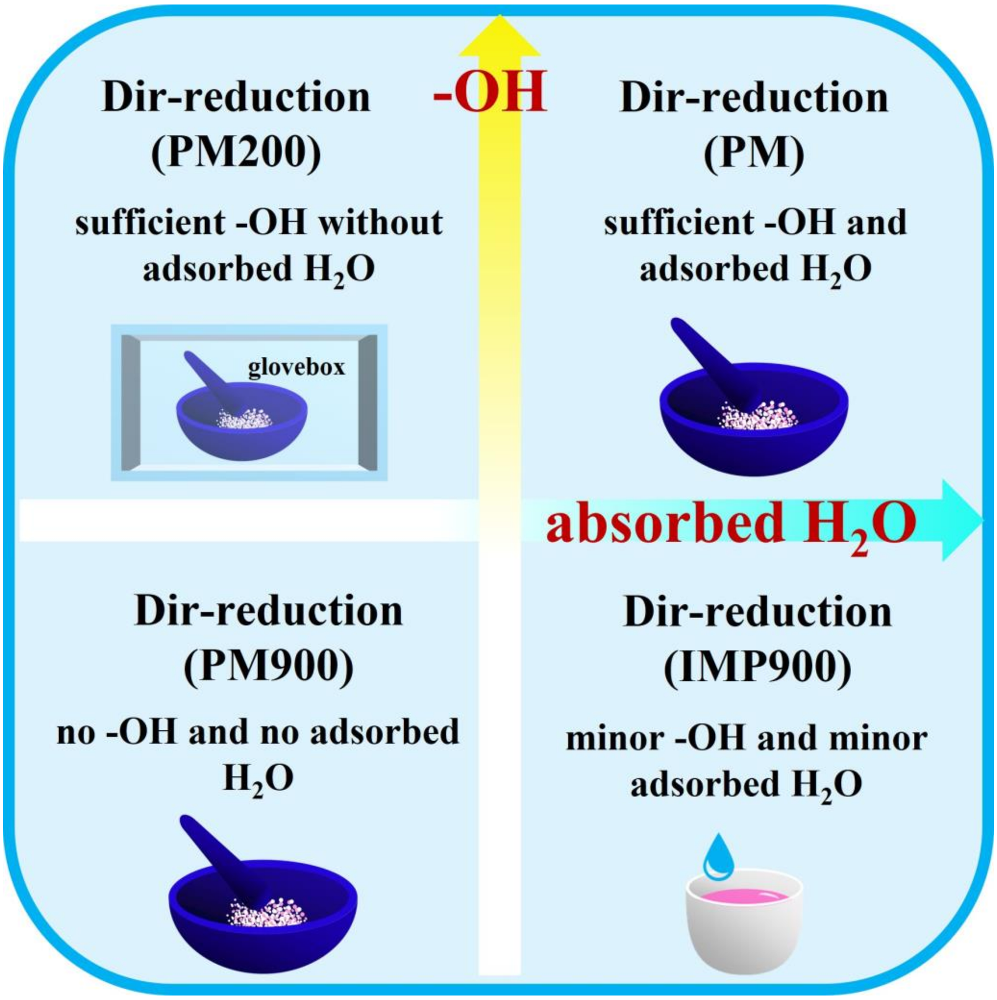


**Supplementary Fig. 12. Relationship between adsorbed water and hydroxyl in four design experiments.**

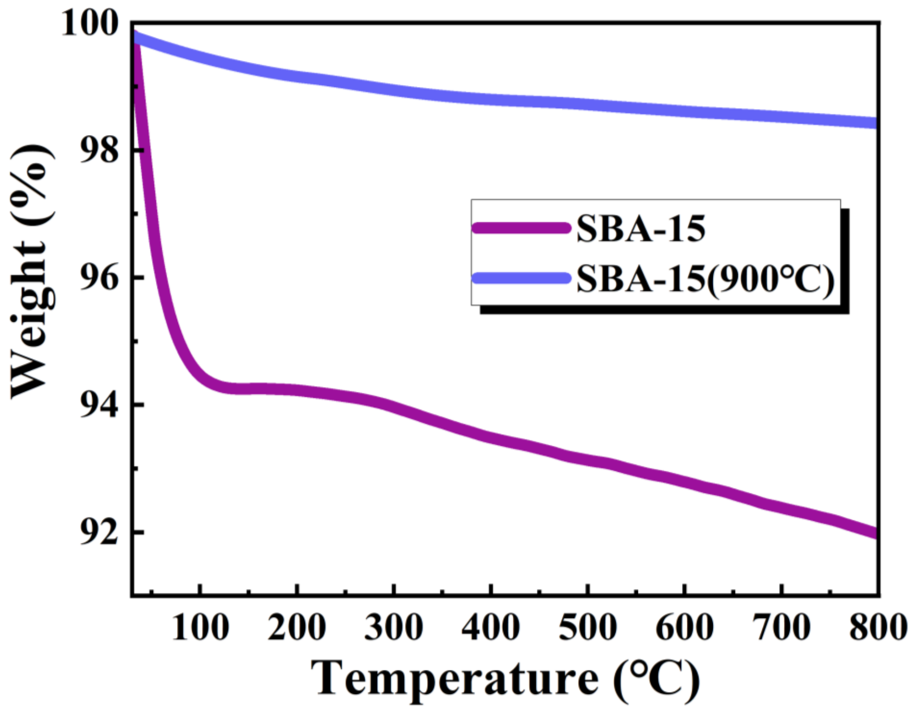


**Supplementary Fig. 13. TG analysis of pure SBA-15 and 900 ºC-calcined SBA-15 under air condition.**

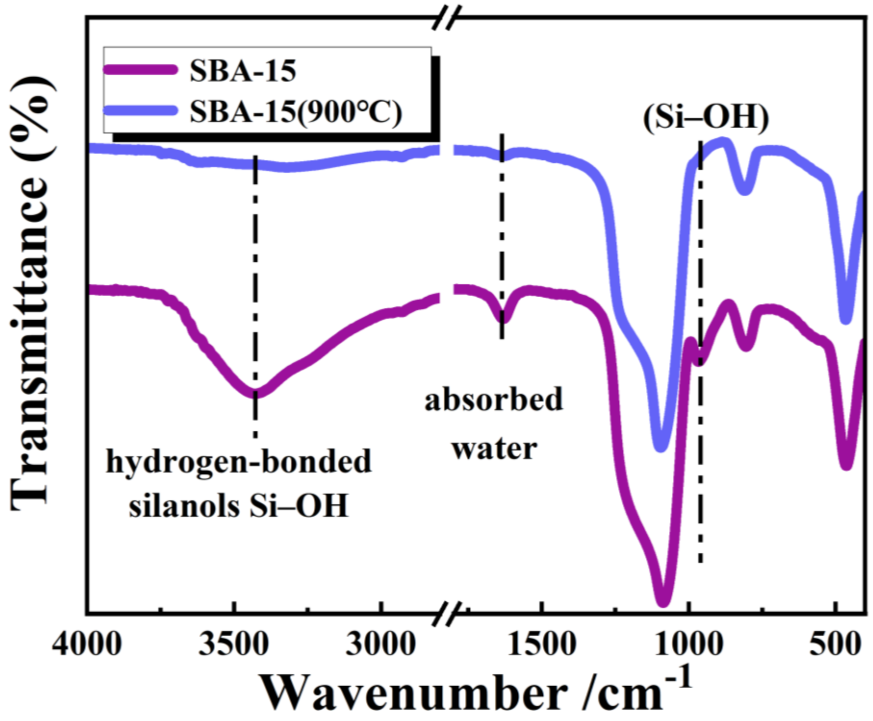


**Supplementary Fig. 14. DRIFTS spectra of SBA-15 and 900 ºC-calcined SBA-15.**

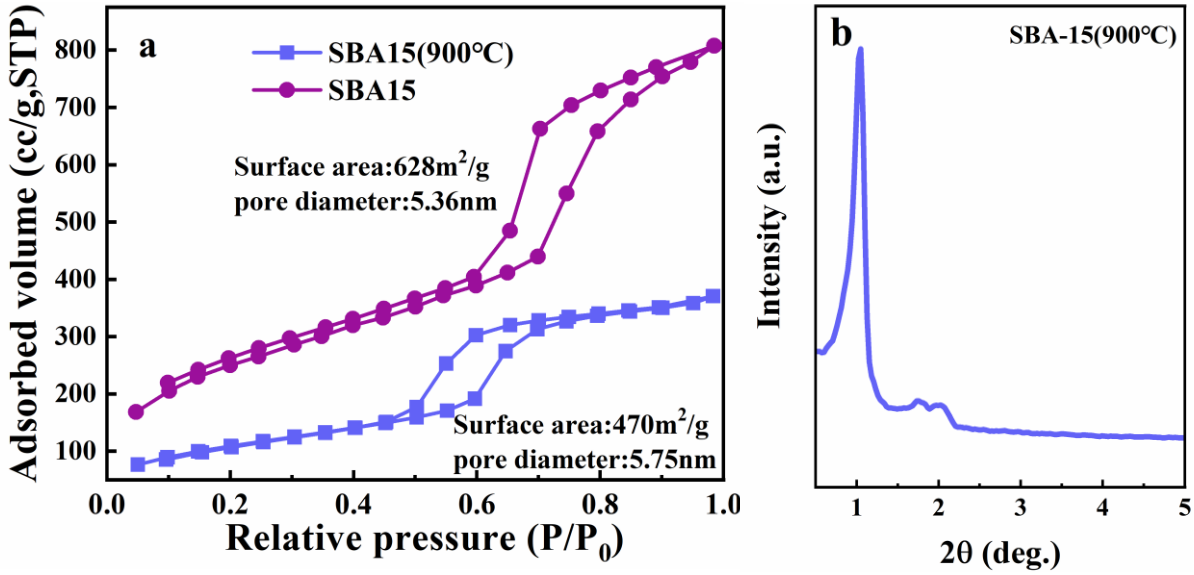


**Supplementary Fig. 15. Physical property of SBA-15** (a) $N_2$ adsorption-desorption isotherms of SBA-15 and SBA-15 (900 $^{o}$C), (b) small angle XRD patterns of SBA-15 (900 $^{o}$C).

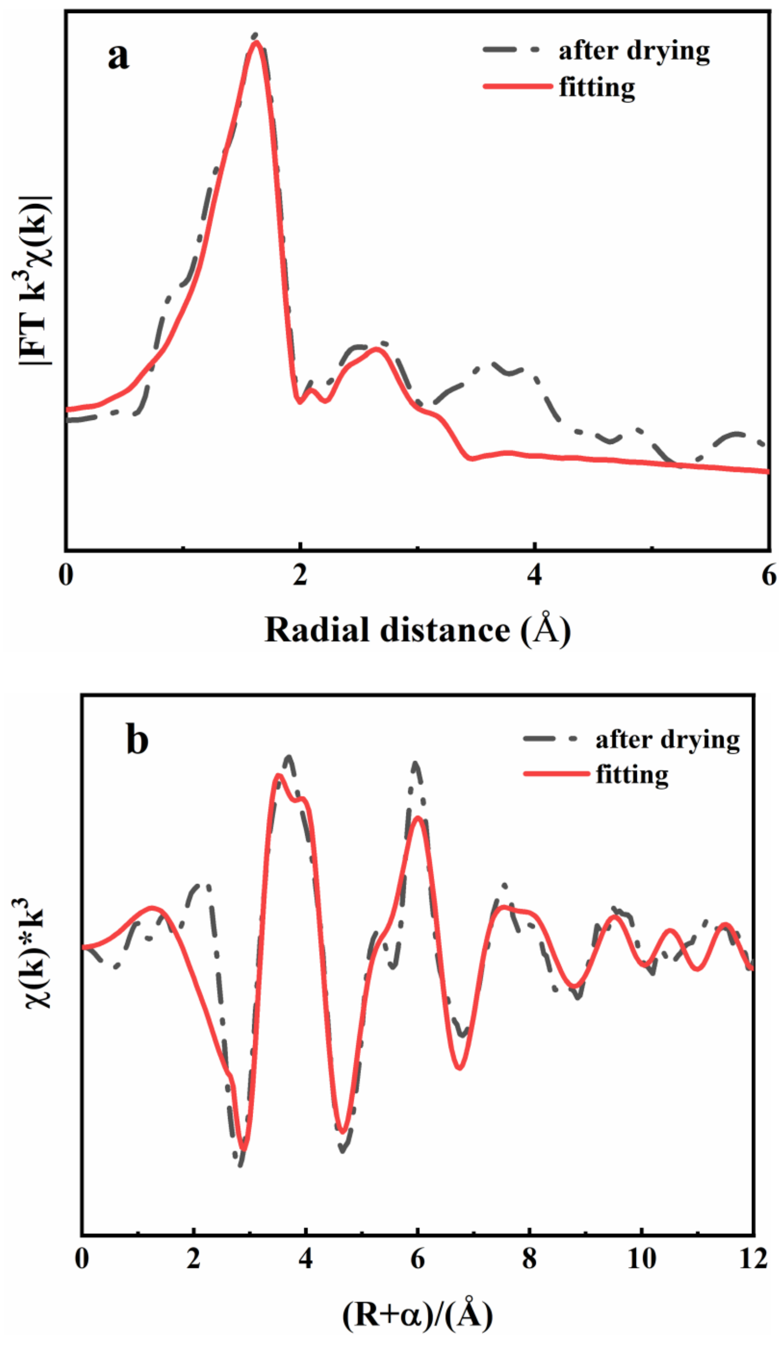


**Supplementary Fig. 16. Fourier transform of the EXAFS spectra.** (a) Fourier transform (FT) $k^3$-weighted$\chi$(k)-function of the EXAFS spectra, and (b) R-space fitting curves for Dir-reduction catalyst.

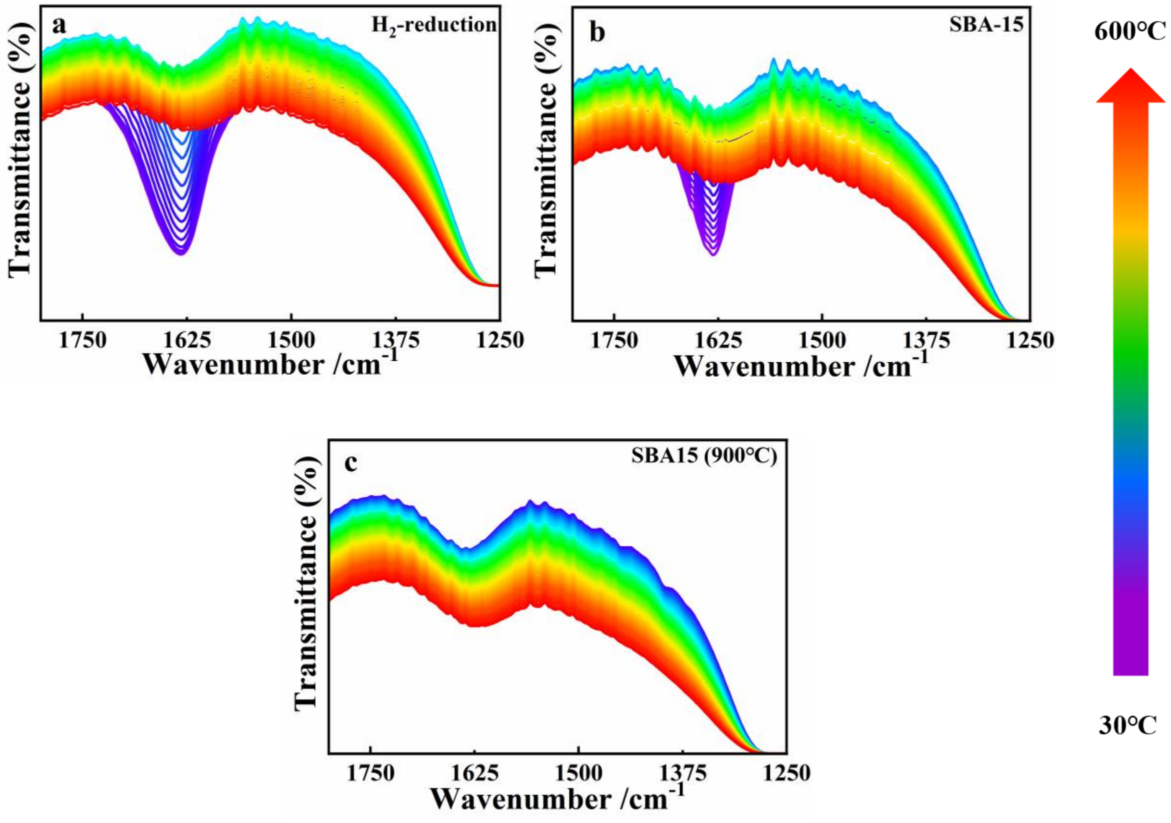


**Supplementary Fig. 17. In-situ FT-IR spectra** of (a) SBA-15, (b) SBA-15 (900 °C), (c) $H_2$-reduction catalyst.

**Supplementary Note 1:** In-situ FT-IR of $H_2$-reduction catalyst, pure SBA-15 and 900 °C-calcined SBA-15 were given in Supplementary Fig. 17. From Supplementary Fig. 17a, no peak related to $NO_3^-$ was exhibited, while the peak of adsorbed water, ascribing to the hydrophilic nature of silica support, was found over the $H_2$-reduction catalyst. Significantly, the peak at 1630 $cm^{-1}$ representing for the adsorbed water was appeared on the SBA-15 (Supplementary Fig. 17b), which was absent on the sample of SBA-15 (900 °C) (Supplementary Fig. 17c). Besides, DRIFTS of SBA-15 and SBA-15 (900 °C) from Supplementary Fig. 14 evidenced that SBA-15 contained

hydrogen-bonded OH, Si-OH and absorbed water, which were corresponding to the peak at 3470 $cm^{-1}$, 960 $cm^{-1}$ and 1630 $cm^{-1}$, respectively[1]. Clearly, these peaks were not existed in the sample of SBA-15 (900 $^{o}C$). Consequently, the above results demonstrated that silica support of SBA-15 tended to easily absorb water in the air, and most of the hydroxyl groups associated with the adsorbed water were removed from SBA-15 after high-temperature calcination (900 $^{o}C$).

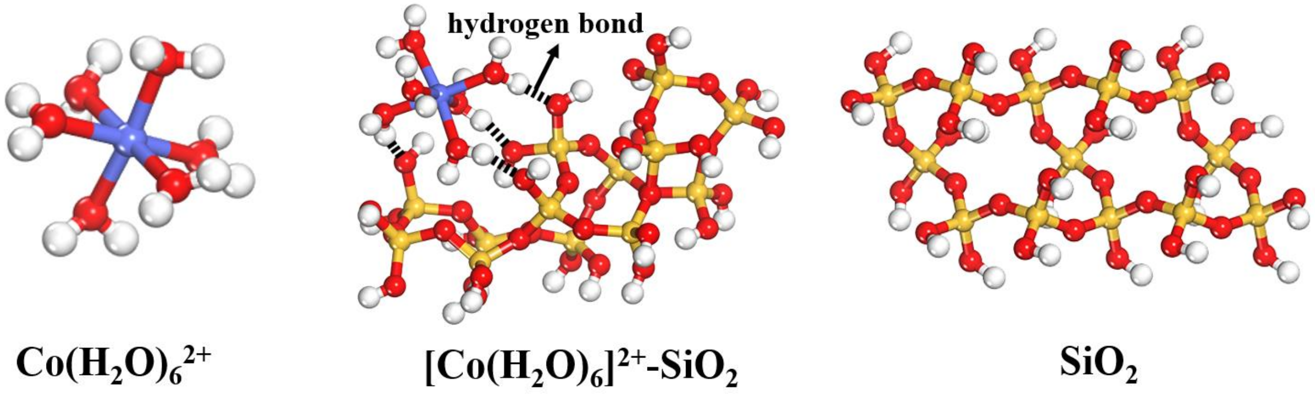


**Supplementary Fig. 18**. **The optimized structure of $[Co(H_2O)_6]^{2+}$, $[Co(H_2O)_6]^{2+}$-$SiO_2$ and $SiO_2$.**

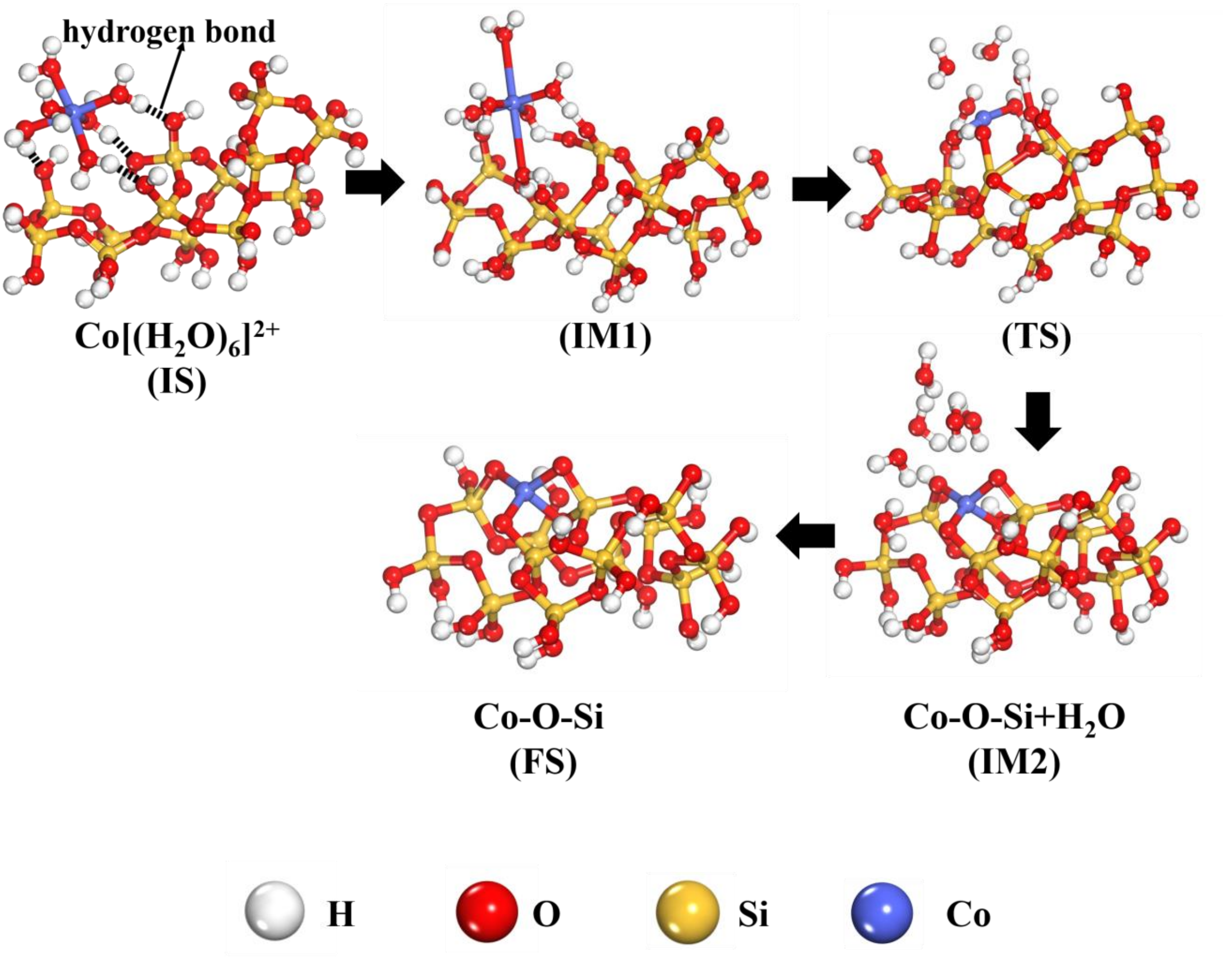


**Supplementary Fig. 19. The flow diagram for the dehydroxylation of hydroxyl groups on Dir-reduction catalyst.**

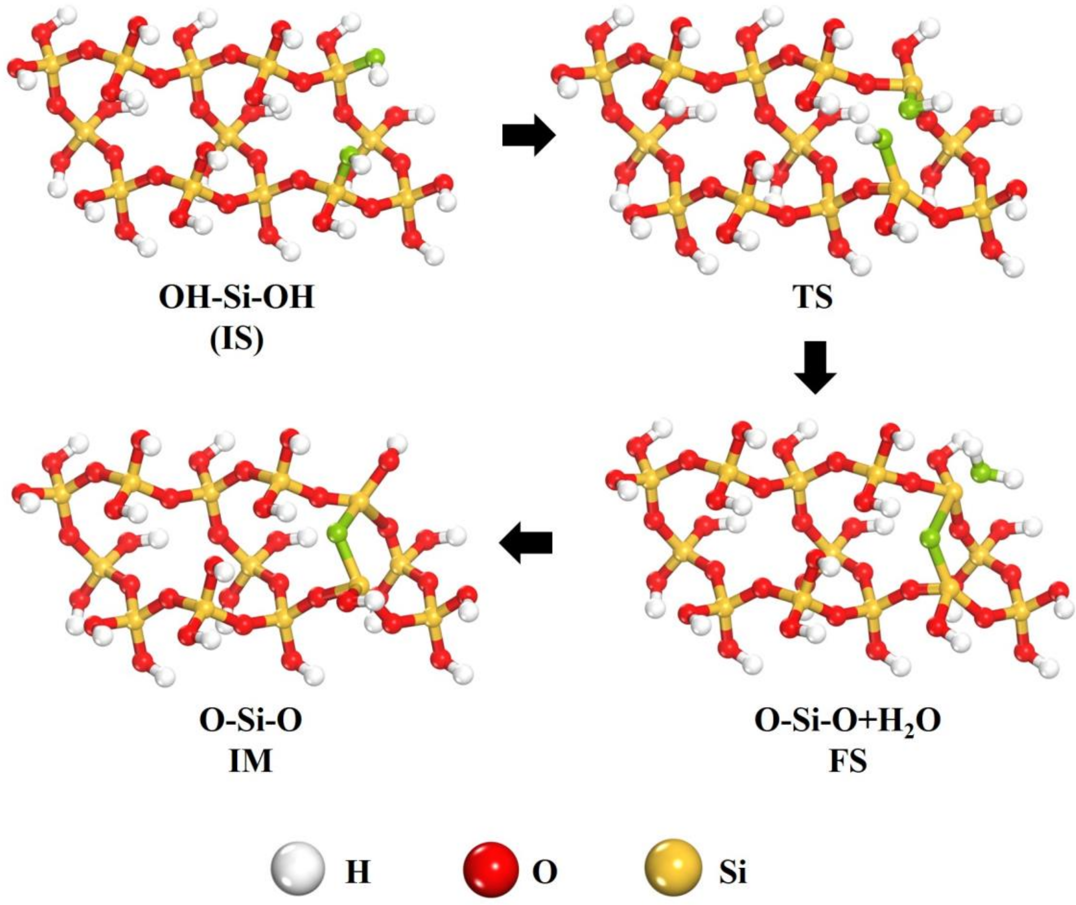


**Supplementary Fig. 20. The flow diagram for the dehydroxylation of hydroxyl groups on the pure SBA-15 catalyst** (To make it easier to show the change of silanol groups, the oxygen was replaced with green atom).

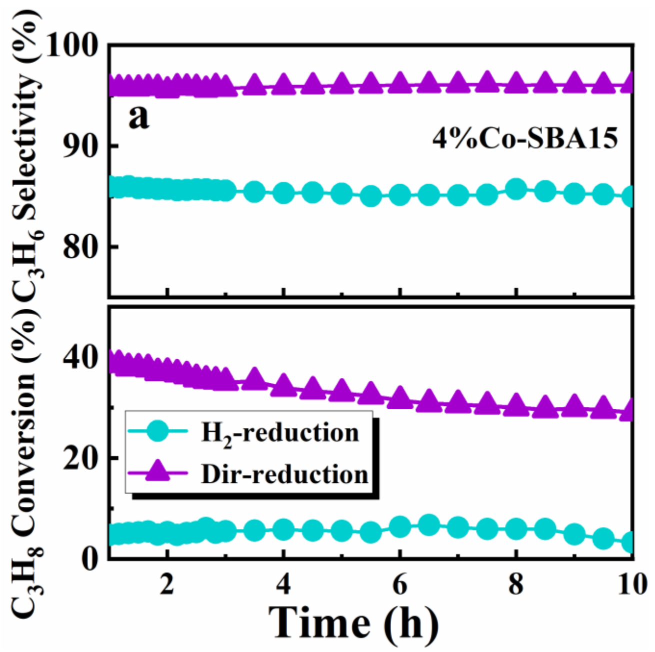


**Supplementary Fig. 21. $C_3H_8$ conversion and $C_3H_6$ selectivity** over (a) 4% Co-SBA-15, (b) 6% Co/SBA-15 from $H_2$-reduction and Dir-reduction processes.

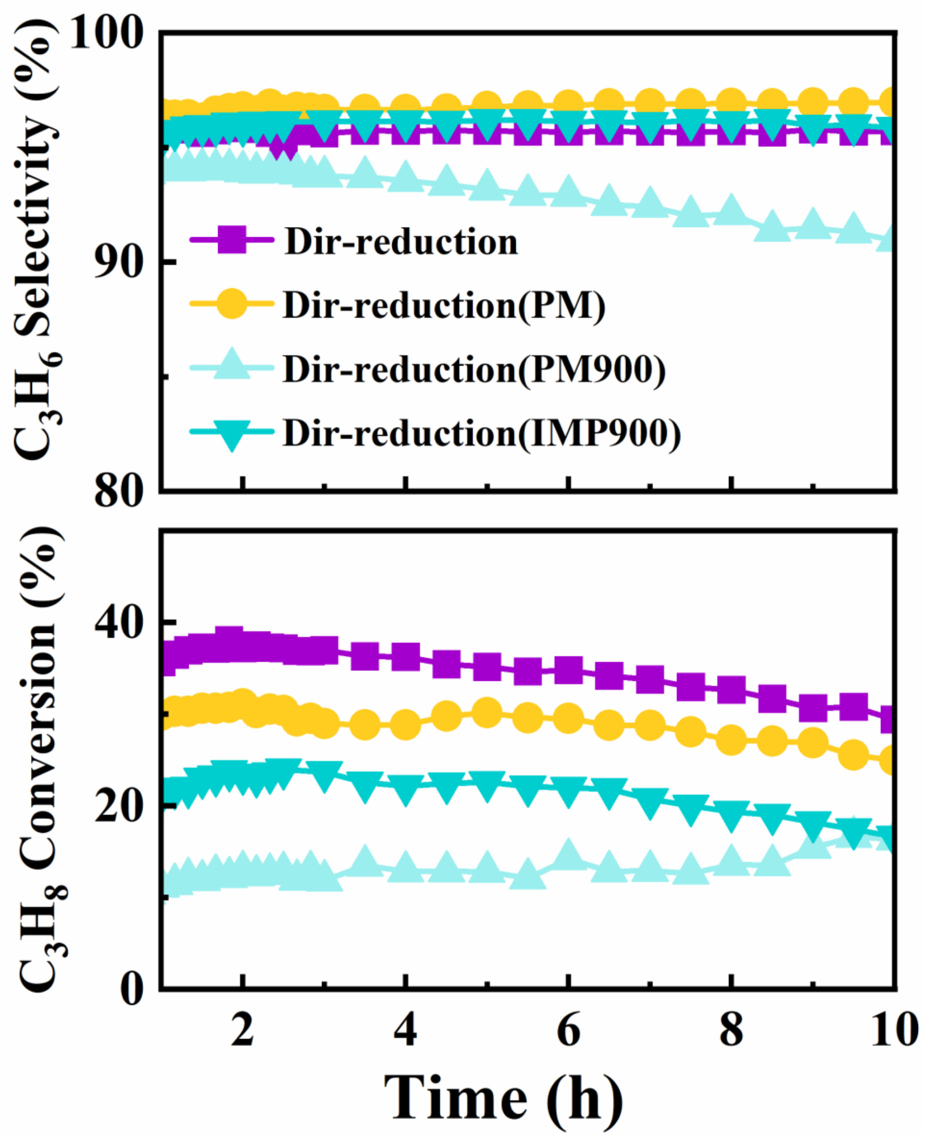


**Supplementary Fig. 22. $C_3H_8$ conversion and $C_3H_6$ selectivity** over Dir-reduction, Dir-reduction (PM), Dir-reduction (PM900) and Dir-reduction (IMP900) catalysts.

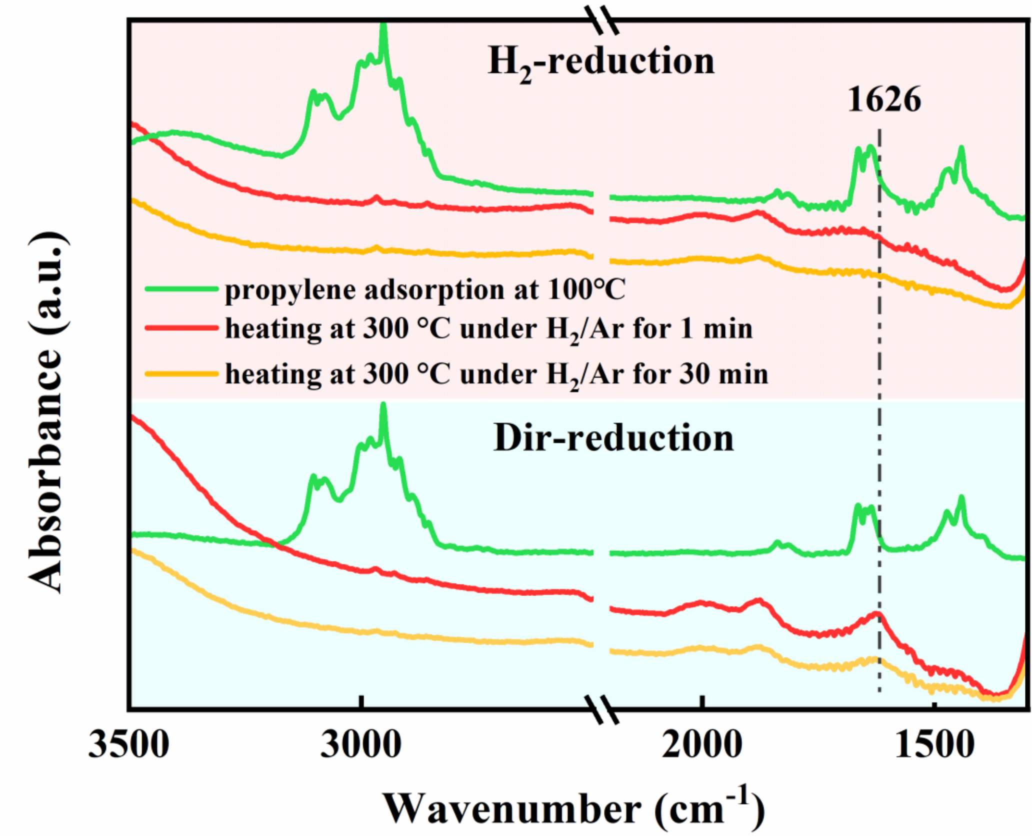


**Supplementary Fig. 23. In-situ FT-IR of propylene hydrogenation process.**

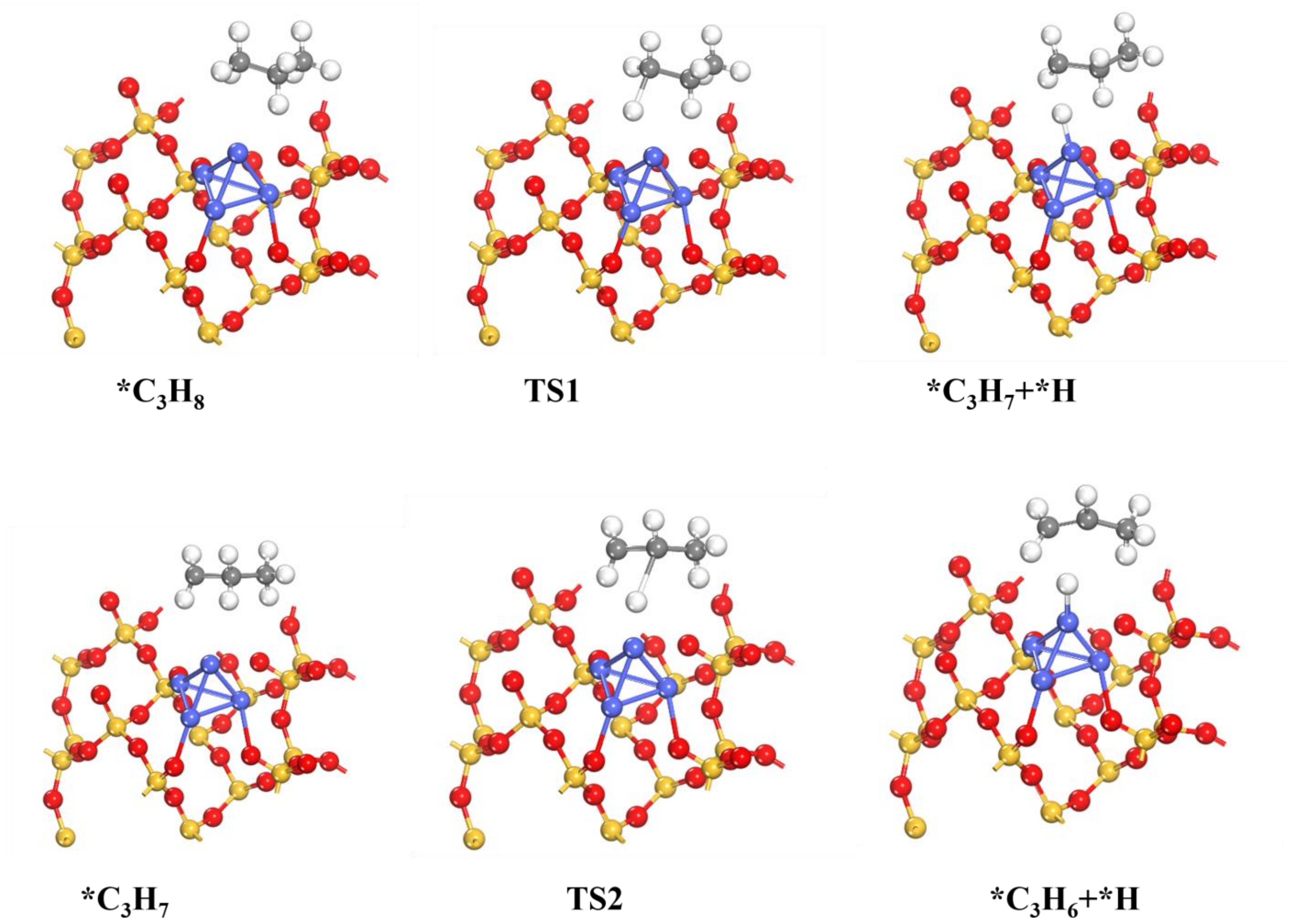


**Supplementary Fig. 24. Mechanism of propane dehydrogenation on $H_2$-reduction catalyst surface.**

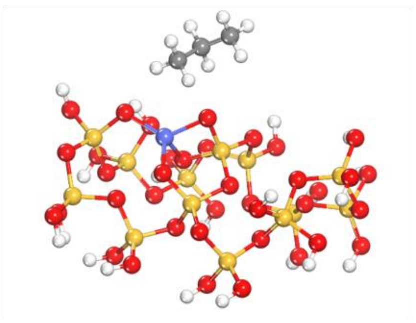

**$*C_3H_8$**

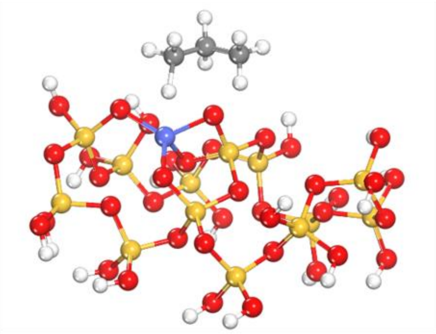

**TS1**

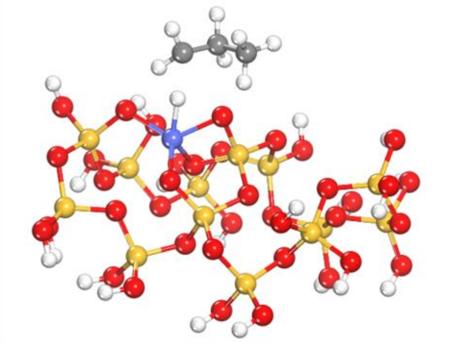

**$*C_3H_7+*H$**

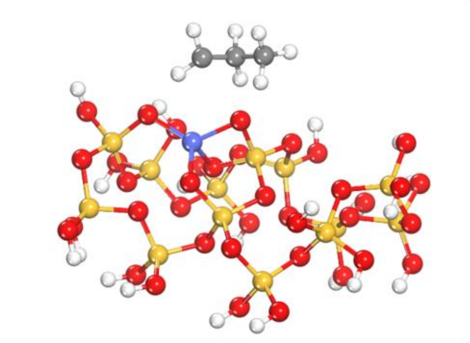

**$*C_3H_7$**

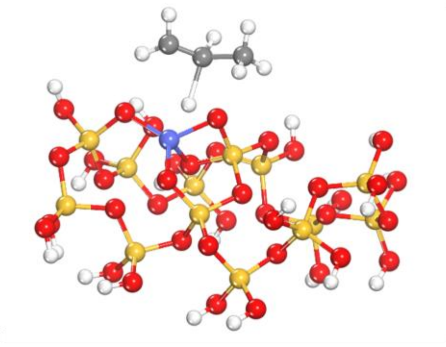

**TS2**

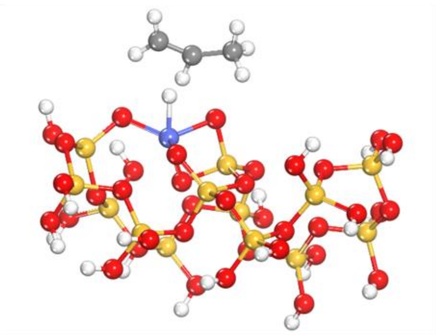

**$*C_3H_6+*H$**

**Supplementary Fig. 25. Mechanism of propane dehydrogenation on Dir-reduction catalyst surface.**

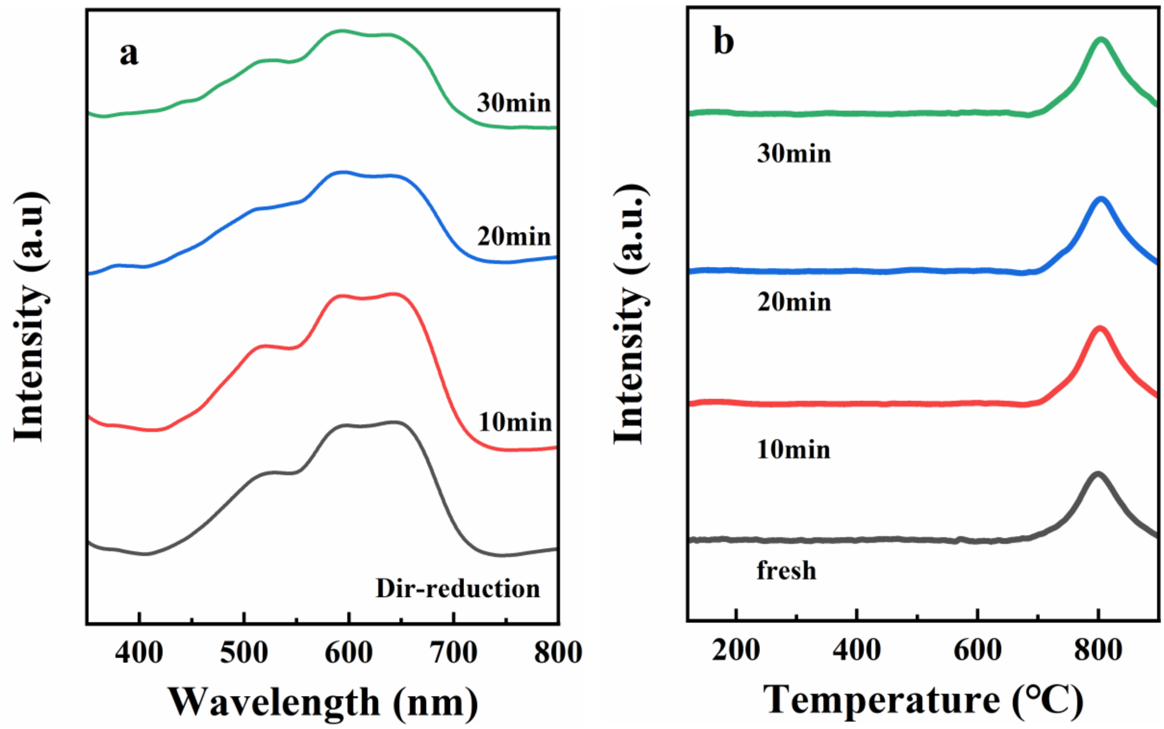


**Supplementary Fig. 26. Characterization of Dir-reduction catalysts during the first 30 min of reaction.** (a) UV-vis spectra and (b) $H_2$-TPR profiles

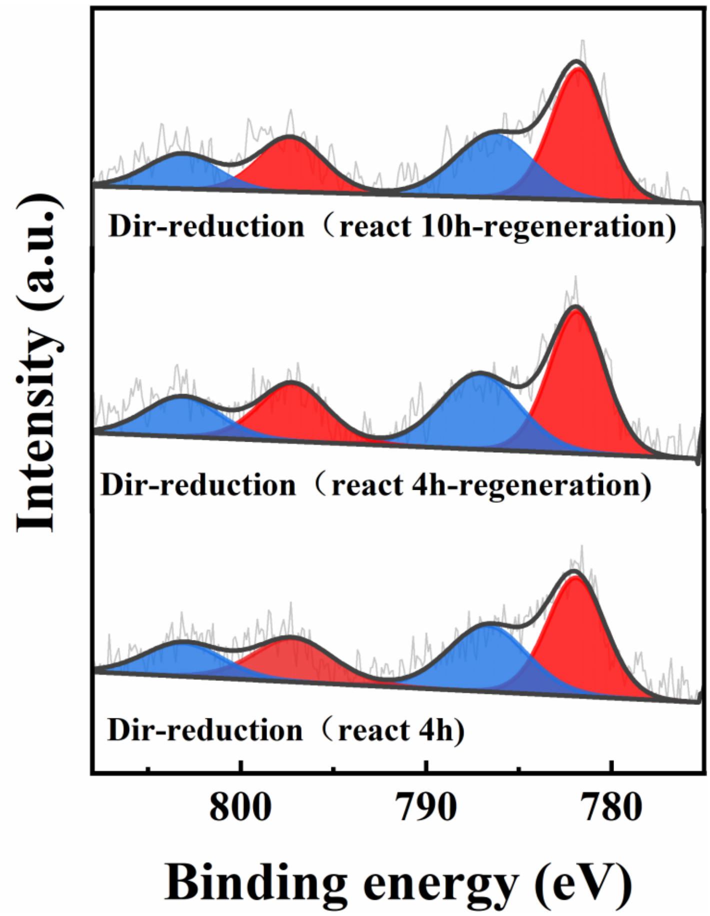


**Supplementary Fig. 27. Ex-situ XPS Co 2*p* spectra** of the regenerated Dir-reduction catalyst.

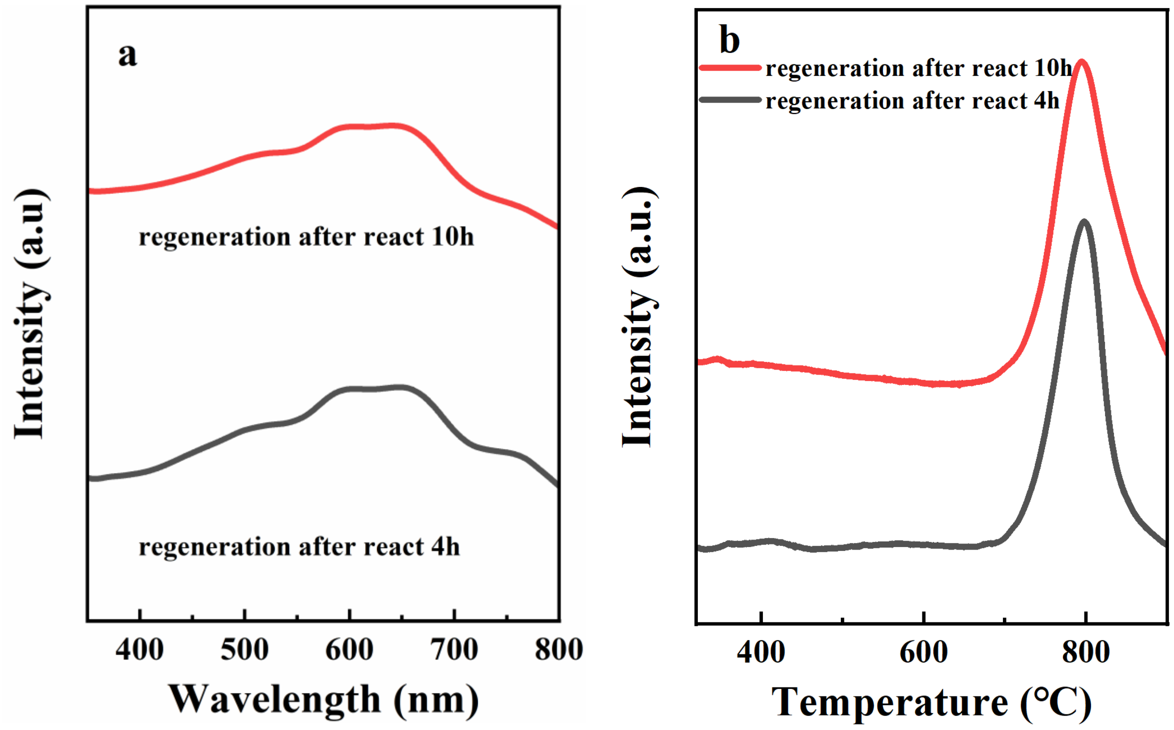


**Supplementary Fig. 28. Characterization of the regenerated Dir-reduction catalyst** (a) UV-vis spectra and (b) $H_2$-TPR profile

## 3. Supplementary Tables

**Supplementary Table 1. EXAFS fitting parameters at the Co K-edge for various samples.**

| Sample | Shell | N [a] | R (Å) [b] | $\sigma^2$ (Å2·$10^{-3}$) [c] | $\Delta E_0$ (eV) [d] | R factor (%) |
|---|---|---|---|---|---|---|
| Co foil | Co-Co | 12* | 2.48±0.003 | 6.12±0.3 | 6.83±0.54 | 0.17 |
| CoO | Co-O | 4* | 1.96±0.039 | 4.49±4.53 | 2.88±2.84 | 1.7 |
| | Co-Co | 12* | 3.10±0.014 | 6.77±1.17 | 6.44±2.07 | |
| $Co_3O_4$ | Co-O | 6* | 1.92±0.014 | 3.60±1.61 | 1.20±1.80 | 0.9 |
| | Co-Co1 | 6* | 2.87±0.022 | 1.41±1.40 | 4.58±5.03 | |
| | Co-Co2 | 6* | 3.33±0.028 | 2.23±1.57 | 4.07±5.02 | |
| Dir-reduction | Co-O | 4.0±0.7 | 2.072±0.009 | 8.12±3.69 | -5.634±1.070 | 0.76 |
| | Co-Co | 4.8±1.6 | 3.121±0.052 | 3.46±2.88 | 5.191±4.322 | |
| Catalyst precursor (after impregnation) | Co-O1 | 5.5±0.9 | 2.089±0.008 | 1.23±2.84 | 1.820±0.955 | 0.53 |
| | Co-N | 5.1±1.2 | 3.083±0.035 | 3.86±2.35 | 8.839±2.747 | |
| | Co-O2 | 6.0±1.8 | 3.514±0.036 | 4.57±2.26 | 3.749±2.522 | |

[a] *N*: coordination numbers; [b] *R*: bond distance; [c] $\sigma^2$: Debye-Waller factors; [d] $\Delta E_0$: the inner potential correction. *R* factor: goodness of fit.

$S_0^2$ was set as 0.95 for Co data, which was obtained from the experimental EXAFS fit of Co foil reference by fixing CN as the known crystallographic value and was fixed to all the samples.
